# Aging: What We Can Learn From Elephants

**DOI:** 10.3389/fragi.2021.726714

**Published:** 2021-08-26

**Authors:** Daniella E. Chusyd, Nicole L. Ackermans, Steven N. Austad, Patrick R. Hof, Michelle M. Mielke, Chet C. Sherwood, David B. Allison

**Affiliations:** ^1^ Department of Epidemiology and Biostatistics, Indiana University-Bloomington, Bloomington, IN, United States; ^2^ Nash Family Department of Neuroscience and Friedman Brain Institute, Icahn School of Medicine at Mount Sinai, New York, NY, United States; ^3^ Center for Anatomy and Functional Morphology, Icahn School of Medicine at Mount Sinai, New York, NY, United States; ^4^ Department of Biology, University of Alabama at Birmingham, Birmingham, AL, United States; ^5^ Nathan Shock Center, University of Alabama at Birmingham, Birmingham, AL, United States; ^6^ Division of Epidemiology, Department of Quantitative Health Sciences and Department of Neurology, Mayo Clinic, Rochester, MN, United States; ^7^ Department of Anthropology and Center for the Advanced Study of Human Paleobiology, George Washington University, Washington, DC, United States

**Keywords:** elephant, animal model, senescence, aging, comparative aging research, gerontology

## Abstract

Elephants are large-brained, social mammals with a long lifespan. Studies of elephants can provide insight into the aging process, which may be relevant to understanding diseases that affect elderly humans because of their shared characteristics that have arisen through independent evolution. Elephants become sexually mature at 12 to 14 years of age and are known to live into, and past, their 7^th^ decade of life. Because of their relatively long lifespans, elephants may have evolved mechanisms to counter age-associated morbidities, such as cancer and cognitive decline. Elephants rely heavily on their memory, and engage in multiple levels of competitive and collaborative relationships because they live in a fission-fusion system. Female matrilineal relatives and dependent offspring form tight family units led by an older-aged matriarch, who serves as the primary repository for social and ecological knowledge in the herd. Similar to humans, elephants demonstrate a dependence on social bonds, memory, and cognition to navigate their environment, behaviors that might be associated with specializations of brain anatomy. Compared with other mammals, the elephant hippocampus is proportionally smaller, whereas the temporal lobe is disproportionately large and expands laterally. The elephant cerebellum is also relatively enlarged, and the cerebral cortex is highly convoluted with numerous gyral folds, more than in humans. Last, an interesting characteristic unique to elephants is the presence of at least 20 copies of the *TP53* tumor suppressor gene*.* Humans have only a single copy. *TP53* encodes for the p53 protein, which is known to orchestrate cellular response to DNA damage. The effects of these multiple copies of *TP53* are still being investigated, but it may be to protect elephants against multiple age-related diseases. For these reasons, among others, studies of elephants would be highly informative for aging research. Elephants present an underappreciated opportunity to explore further common principles of aging in a large-brained mammal with extended longevity. Such research can contribute to contextualizing our knowledge of age-associated morbidities in humans.

## Introduction

Aging is a complex and multifaceted process with species-specific characteristics. Differences among species can be informative, and have the potential to reveal the causes and consequences of aging across variation in life history, ecology, and phylogeny (Austad, 1997). Instructive species for comparative investigation include those that share relevant characteristics with humans (Austad, 2009). Elephants are particularly fascinating in this regard.

Like humans, elephants are K-selected animals: they generally give birth to single offspring, have slow maturation, and long lifespans. Elephants are the second longest living terrestrial mammal, behind only humans (Moss, 2001; Sukumar, 2003; Wittemyer et al., 2013; Turkalo et al., 2018). In fact, in a nearly 50 years study of wild elephants in East Africa, the life expectancy of female elephants at birth (mean, 46.7 years) was reported to exceed that of Hadza hunter-gatherers living nearby (35.6 years) (Moss et al., 2011; Blurton Jones, 2016). Elephants are one of the slowest reproducing terrestrial mammals, with the longest gestational period (Moss, 1983) and have an age of sexual maturity and an interbirth interval comparable to those of humans (Moss, 2001; Wittemyer et al., 2013) (Table 1). They have one of the largest proportional postmaturation to prematuration ratios, possibly surpassing that of humans. It is not only elephants’ general traits relative to aging, but their cognitive abilities, emotional complexity, and strong social ties that make them especially appealing for comparative investigation.

**TABLE 1:** Comparative life history of the Amboseli, Kenya, African savanna elephants and Hadza hunter-gatherers.

	African savanna elephant Moss et al. (2011)	Hadza hunter-gatherers Blurton Jones (2016)
Age at first birth (mean in years) (range)	14 (9–22)	19 (14–27)
Age at last birth (mean in years)	Majority continue to reproduce throughout life	37
Gestation (months)	22	9
Interbirth interval (years)	4.5	3.5
Mean life expectancy at birth, natural mortality only years)^a^	Female: 46.7	Female: 35.55
	Male: 37.4	Male: 30.81
Mean life expectancy at age 20, natural mortality only (roughly sexual maturity; years)^a^	Female: 37.9	Female: 41.75
	Male: 31.1	Male: 36.27
Maximum lifespan (years)	Female: >65	Female: 86
	Male: ∼ 60	Male: 83

a The reason that elephants have a higher life expectancy compared to Hadza hunter-gathers at birth is because the Hadza experienced higher infant mortality.

Although humans and elephants share many characteristics of interest, they are not evolutionarily closely related. The separate lineages leading to elephants and humans diverged approximately 100 million years ago (mya). Thus, studying elephant aging in a comparative perspective may reveal key physiologic mechanisms associated with aging. For example, it may prove beneficial to investigate whether elephants develop age-related diseases to the same extent as humans, when living to comparable ages. This is of particular relevance as age-associated diseases are a growing public health issue, and one major limitation of ongoing therapeutic research is the lack of animal models that accurately translate to people. To address this concern, newer, and possibly more appropriate models, like elephants, are needed. Thus, emphasizing the importance of the comparative perspective, the aim of this article is to highlight the unique properties of elephants and why studying them can push aging research forward.

## Evolution and Longevity

The family Elephantidae was once a flourishing group of the order Proboscidea, living throughout much of Africa, Eurasia, and the Americas (Shoshani, 1998). Only three species still survive: the African savanna (*Loxodonta africana*), African forest (*Loxodonta cyclotis*), and Asian (*Elephas maximus*) elephants (Gobush et al., 2021). Elephants evolved from an ancestry within the afrotherian clade (which also includes manatees, hyraxes, aardvarks, tenrecs, elephant shrews, and golden moles) (Glickman et al., 2005). Mitochondrial DNA analyses suggest that elephants (as well as manatees and hyraxes) have a common aquatic ancestor (de Jong, 1998; Mirceta et al., 2013). African elephants diverged from the lineage leading to Asian elephants (and mammoths) approximately 7.6 mya, while African savanna and forest elephants diverged approximately 4.0 mya (Rohland et al., 2007).

One of the defining characteristics of all extant elephants is their longevity. Wild savanna and forest elephants, and zoo and semi-captive Asian elephants are known to live into their 7th decade of life (Lee et al., 2012; Keele, 2014; Lahdenperä et al., 2014; Chapman et al., 2019), with some Asian elephants documented to live into almost their 80s (Lahdenperä et al., 2014). Wild elephants have been living to advanced ages for millennia, without the aid of science or medicine. Such longevity is rare in any terrestrial mammal, which suggests that elephants have evolved mechanisms to protect against aging diseases.

In fact, it is possible that changes in gene expression due to DNA methylation can be used as a marker of longevity with potential mechanistic influence. This line of thinking has led to the recent development of various epigenetic clocks to measure biological age. One of the most commonly applied epigenetic clocks (the Horvath clock) was recently used to examine the rate of accumulation of DNA methylation marks in savanna and Asian elephants, and to create a dual human-elephant clock (Prado et al., 2021). Interestingly, most CpGs demonstrate opposite aging effects between humans and elephants, including genes associated with respiratory system processes, circadian rhythms, mitochondrial function, and some cancer-related signatures (Prado et al., 2021). Researchers have also conducted lifespan estimates of extinct Elephantidae species. For example, by using a lifespan clock, the lifespan of the woolly mammoth (*Mammuthus primigenius*) and that of the straight-tusked elephant (*Palaeoloxodon antiquus*) were estimated to be 60 years (Mayne et al., 2019), similar to what is observed in the extant elephant species.

Considering their long lifespan, the age at which female and male elephants start reproducing is relatively late. Savanna and Asian female elephants may start to conceive at 11–14 years of age and give birth every 3–4 years (Moss et al., 2011). Forest elephants appear to start reproducing later in life, at 20 years of age on average, with interbirth intervals of every 5–6 years (Turkalo et al., 2017). Because little is known about forest elephants, it is unclear whether their comparatively delayed primiparous age is representative of forest elephants in general or is specific to this particular population studied. Nevertheless, while some female Asian elephants may experience an extended post-reproductive stage (Chapman et al., 2019), females from all three species are capable of reproducing into their 60s (Moss, 2001; Lahdenperä et al., 2014; Turkalo et al., 2018).

Males have a unique combination of behavioral and physiologic traits that reflect the intense pressure to compete for access to estrous females [in general, females are in estrous for only 3–6 days every 3–9 years, see (Blurton Jones, 2016; Mayne et al., 2019) for review]. Males grow throughout much, or perhaps all, of their lifespan, in terms of stature, as well as body and tusk weight (Roth, 1984; Haynes, 1993; Lindeque and Jaarsveld, 1993; Lee and Moss, 1995). Males experience musth, unique to elephants, which is characterized by bouts of elevated testosterone and aggression, and heightened sexual activity. Females prefer larger males and those in musth, which may explain why paternity success steadily increases in males from the mid-20s until it peaks around early 50s, after which, it is comparable to a male in his early 40s (Hollister-Smith et al., 2007). This observation suggests male elephants may undergo sexual selection for longevity.

One mechanism allowing elephants to reach longer lifespans may be their multiple copies of the tumor suppressor gene *TP53* (Abegglen et al., 2015; Sulak et al., 2016), colloquially known as the “guardian of the genome.” Humans have one copy of *TP53*, whereas savanna, forest, and Asian elephants are estimated to have 19–23, 21–24, and 19–22 *TP53* copies, respectively (Tollis et al., 2020). This is compared to estimates of 19–28, and 22–25 *TP53* copies in the extinct woolly mammoth and straight-tusked elephant, respectively (Tollis et al., 2020). Other afrotherian species, such as the manatee and rock hyrax, have two copies of *TP53*, while Bowhead and Minke whales each have one, respectively (Sulak et al., 2016). Of the multiple elephant *TP53* genes, only one appears to have a comparable gene structure to other mammals, while the other copies appear to be retrogenes, as they lack true introns (Abegglen et al., 2015). Retrogenes can have functional biological roles (Pink et al., 2011). Indeed, genetic variation at some elephant *TP53* retrogenes is conserved across all three extant elephant species, providing evidence of the functionality of at least some *TP53* retrogenes (Vazquez et al., 2018; Tollis et al., 2020), and functional *TP53* duplicates appear to occur only in the elephant lineage (and possibly some bats) (Sulak et al., 2016). p53 (encoded by the *TP53* gene) is a transcription regulator [reviewed in (Laptenko and Prives, 2006)]. When DNA is damaged, p53 can cause cell-cycle arrest, senescence, or apoptosis and/or it can stimulate DNA repair, thereby promoting removal or repair of damaged cells [reviewed in (Williams and Schumacher, 2016)] and suppressing tumors.

As reported recently, *TP53* is activated in response to cellular stresses in addition to DNA damage (Haupt and Haupt, 2017). Thus, these multiple copies may have various effects in response to cell stress (Kastenhuber and Lowe, 2017). Elephants appear to have an enhanced apoptotic response to DNA damage owing to their extensive number of *TP53* (*EP53*) retrogenes (Abegglen et al., 2015; Sulak et al., 2016) and, as a result, develop cancer at lower rates than expected for their body size and lifespan (Abegglen et al., 2015; Tollis et al., 2020). Interestingly, Asian elephants appear to develop benign tumors and malignant cancer at higher rates than do savanna elephants (Tollis et al., 2020). Because cancer is an age-related disease, the prevalence is significant in the context of the evolution of extended longevity (Lemaître et al., 2020). Thus, long life requires a delay or decrease in cancer occurrence, in addition to a reduction of other aging pathologies (Lucas and Keller, 2020). In addition to its involvement in cancer, p53 has other relevant associations, including its association with Alzheimer’s disease (AD), and its central role in aging. Thus, elephants provide a unique opportunity to further investigate the potential protective effects of p53 in not only cancer, but aging in general.

## Complex Social Bonds, Memory, and Cognitive Ability

Elephants societies are a fluid, fission-fusion system, such that group members change daily or seasonally (Moss and Poole, 1983; Wittemyer et al., 2005). At the center of elephant society is the family, comprised of female matrilineal relatives and dependent offspring. The tight-knit family members demonstrate remarkable cooperation, moving, foraging, and making decisions together. Families at times join together to form bond groups, and occasionally form an additional social tier termed clans (Wittemyer et al., 2005). Families are led by a matriarch, who is the primary repository for social and ecological knowledge. Matriarchs are largely responsible for the survival of their whole family. Families with older-aged matriarchs are overall more successful, in terms of both survival and reproduction. Calves are dependent on their mothers and other family members for social support, survival, and learning, constantly being touched, guided, and reassured throughout the first years of life. While females remain with their natal herd, usually for life, males depart at an average age of 14 years (Moss et al., 2011), after which they will join small, all-male groups, albeit with looser arrangements than the females (Murphy et al., 2020). Similar to other social species, such as humans and free-living populations of baboons (Holt-Lunstad et al., 2010; Silk et al., 2010), sociality and longevity appear to be positively related in elephants. In addition to being critical for family survival, the oldest females (the matriarchs) provide protection for calves, with higher calf survival in families with grandmothers, and they maintain the social cohesion within the herd (Moss et al., 2011). Behavioral aging, characterized by cognitive decline and social isolation, does not appear to be common in elephants (Lee et al., 2016).

Elephants have evolved to rely heavily on their cognitive abilities. Data support that living in socially intricate networks correlates with, and likely encourages, greater cognitive skills (Bates and Byrne, 2007). Unquestionably, elephants excel in long-term, spatiotemporal, and social memory. Evidence from both Asian and African elephant ethological research suggests that elephants likely have strong spatial and episodic memories. They appear to navigate complex physical and social environments over hundreds of miles using direct and indirect experience (Jacobson and Plotnik, 2020). Other research has shown that elephants retain long-term memory of reward stimuli (Markowitz et al., 1975), can identify and locate more than 100 out-of-view family members (Bates et al., 2008), and can spatially locate waterholes over 100 km distances and extended periods of time (more than 3 years) (Polansky et al., 2015). Elephants appear to have learned to discern between human ethnic groups that vary in their level of threat toward elephants (Bates et al., 2007; McComb et al., 2014). Elephants also seem to be unique among non-human animals in that they may exhibit behaviors related to “theory of mind”, demonstrating self-awareness (Plotnik et al., 2010), cooperation with one another (Plotnik et al., 2011), mourning-like behavior (Goldenberg and Wittemyer, 2020), empathy (Byrne et al., 2008), and consolation (Plotnik and de Waal, 2014).

Elephants’ remarkable long-term memory and strong social ties appear to leave them susceptible to psychological trauma. Wild elephant populations have experienced high levels of violence (i.e., poaching—elephants killed for their tusks). At the peak of poaching in Africa in 2011, approximately 40,000 elephants were illegally killed in just that year alone, equating to a possible species reduction of 3% (Wittemyer et al., 2014). Although elephants have processes, rituals, and social structures to respond to trauma, including behaviors that resemble grieving, mourning, and socializing, the magnitude and nature of human violence has disrupted elephants’ ability to use these practices, leading to what has been described as post-traumatic stress disorder (PTSD) (Bradshaw et al., 2005). In one example, in South Africa, teenaged orphaned male elephants were uncharacteristically violent, killing over 100 rhinoceroses (an aberrant behavior for elephants) (Bradshaw et al., 2005). In addition, male elephants with PTSD were responsible for 90% of all male elephant deaths in their community, compared with 6% in relatively unstressed communities (Bradshaw et al., 2005). Calves who survive witnessing their mother (and sometimes their entire families) being killed visually demonstrate an emotion akin to despair. It is possible, and something we are currently investigating, that these traumatized orphaned elephants develop health issues later in life and have accelerated aging, similar to children and wild baboons with higher adverse early life experiences (Felitti et al., 1998; Brown et al., 2009; Brown et al., 2010; Tung et al., 2016). Elephants’ dependence on social bonds, memory, and cognition highlights their potential in studying age-related cognitive decline, which may uncover specific adaptations in the wider context of the evolution of cognition.

## Brain Size and Composition

Brain size has been shown to be related to body size, sociality, and lifespan in certain groups of mammals (Street et al., 2017). Species with larger brains (in absolute size and also relative to body size), on average, demonstrate a greater ability to process and use complex information (Holloway et al., 1979; Deaner et al., 2007). Over the course of evolution, the encephalization quotient (EQ, which is a measure of how much larger a species’ brain is than expected by general allometric scaling for a given body size) of *Proboscidea* has increased by 10-fold, to about 2.0 for extant elephants. Thus, the elephant brain is twice as large as would be expected for an average mammal of the same body size (Shoshani et al., 2006). The adult elephant brain averages around 5 kg (Shoshani et al., 2006; Herculano-Houzel et al., 2014), which is the largest among living and extinct terrestrial mammals, and three times the absolute size of the human brain. For large-brained, long-lived species, there is a need to develop improved aerobic energy production to fuel neuronal activity. When comparing protein evolution associated with brain, lifespan, and metabolism between humans and elephants, a convergent pattern is observed (Goodman et al., 2009). Specifically, in comparison to their phylogenetic relatives, elephants and humans have independently evolved and share increased nonsynonymous amino acid substitution rates among nuclear genes that code for mitochondrial proteins that function in aerobic energy metabolism (Goodman et al., 2009). This adaptive evolution of protein structure in elephants and humans likely minimizes reactive oxidative species, helping to reduce DNA damage and preserve long-lived neurons (Goodman et al., 2009).

Because elephants have extensive memory abilities, appearing to exceed those of great apes and possibly even humans, it is important to examine specific anatomical brain structures. For example, the elephant brain’s cerebral gyral pattern is more complex with more gyri than in primates, including humans, and carnivores, but is less complex than in cetaceans (Shoshani et al., 2006), which is a predictable pattern given the cortical surface area and thickness (Mota and Herculano-Houzel, 2015). Elephants have the greatest volume of cerebral cortex, with large, nonprimary areas thought to be involved in higher-order brain functions (Hart and Hart, 2010). The hippocampus, which is crucial for the formation and retention of cognitive maps that code for unfamiliar spatiotemporal relationships (Sweatt, 2004), is comparable in absolute size between elephants and humans, albeit proportionally smaller in elephants (Herculano-Houzel et al., 2014) and has the expected architecture for a mammalian hippocampus (Patzke et al., 2014). The elephant temporal lobe is disproportionately large compared with that of humans and expands laterally (Shoshani et al., 2006). Lastly, the elephant’s cerebellum has deviated markedly in evolution. It is the relatively largest compared to the rest of the brain size of all other mammals, and the lateral cerebellar hemispheres are expanded compared to the vermis (Maseko et al., 2012), although there are mammals that have greater lateral cerebellar relative enlargement (Smaers et al., 2018). The elephant cerebellum is specialized such that it has increased neurons relative to the cerebral cortex [97.5% of the 257 billion neurons in the elephant brain are found in the cerebellum (Herculano-Houzel et al., 2014)], and the neurons are packed more compactly than in other afrotherians (Herculano-Houzel et al., 2014).

Cortical pyramidal neuron morphology also differs between humans and elephants. Human neurons have basal dendritic trees with a greater number of short branches and a vertical apical dendrite, whereas elephants appear to have longer basal dendritic segments and a V-shaped bifurcating apical dendritic arrangement (Jacobs et al., 2011). This suggests potential differences in cortical information processing, possibly what allows elephants to have such an extraordinary long-term, spatiotemporal, and social memory abilities (Hart and Hart, 2010).

In this regard, elephants appear to be quite attractive in the study of AD and neurodegeneration. As alluded to earlier, families with older-aged matriarchs are more successful than families with young matriarchs because they rely on the matriarch’s ability to remember critical spatiotemporal and social information. Thus, it is possible that elephants evolved protective mechanisms to slow neurodegeneration. To our knowledge, however, no published reports address whether elephants develop neuropathologic changes similar to AD or related dementias or even the amount of neurodegeneration that occurs with normal aging in the absence of disease. We have therefore begun to examine and quantify age-related brain changes in elephants.

**FIGURE 1:**
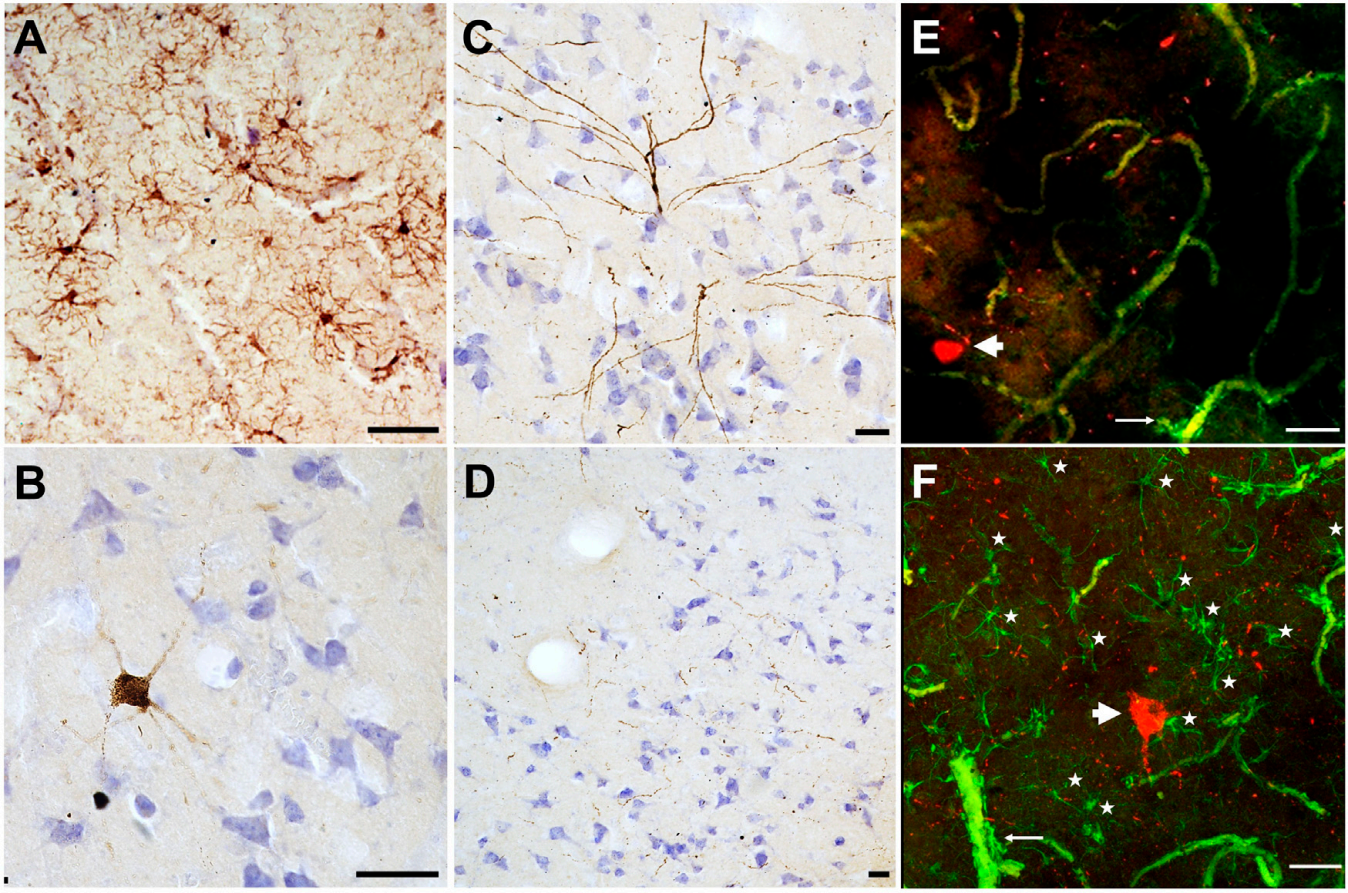
Microglia and phospho-tau detected in brain tissue collected from the cortex of a 51-year-old female Asian zoo elephant (*Elephas maximus*). Tissue stained using immunohistochemistry and counterstained with cresyl violet, **(A)** IBA1 (brown, 1:1,000, Fujifilm, 019–19,741), **(B–D)** CP13 (brown, 1:1,000, Gift from Dr. P. Davies). Images **(A–D)** were taken on an Axiophot brightfield microscope (Carl Zeiss Microscopy, Jena, Germany), with a 10x/0.32 Plan-Apochromat objective. **(E–F)** Tissue was stained with CP13 (red) and GFAP (green, 1:1,000, Abcam., ab68428) using immunofluorescence (note that blood vessels appear in green due to autofluorescence). Images **(E–F)** were taken on a CLSM 780 confocal microscope (Carl Zeiss Microscopy, Jena, Germany), using a 20x/0.8 DICII objective and DPSS 561–10 diode and Argon lasers at excitation wavelengths of 555 and 488 nm. Confocal stacks in layers II and III of the cerebral cortex were imaged at 512 × 512 pixel resolution with a z-step of 1 µm for a pinhole setting of 1 Airy unit. Images are presented as maximum intensity projections of the Z-stack, made using ZenBlue (version 3.3, Carl Zeiss Microscopy, Jena, Germany). All scale bars are 50 μm. In **(A)**, microglia are evenly distributed and ramified without any activated (ameboid) forms. In **(B, C)**, neurons stained with tau were found in layers III and II, respectively. In **(D)** tau “speckling” is visible in brown throughout layer III, and it is also visible in **(E–F)** in red. Images **(E–F)** show tau-positive neurons (thick arrowheads), and fibrils in red, and astrocytes in green (as well as autofluorescent blood vessels which are much thicker). Astrocytic end feet are visible around some blood vessels (thin arrows). In **(F)**, astrocytes are indicated with stars, note their presence around the tau-positive neuron.

In our preliminary work, we sampled brain tissue from the prefrontal cortex of a 51-year-old female Asian zoo elephant for immunohistochemistry and immunofluorescence. The tissue was stained with ionized binding adaptor molecule-1 (Iba-1), a calcium binding protein specific to microglia and macrophages to detect any differences in microglia morphology. Microglia can clump together and change shape to become less ramified when activated in response to neurodegeneration (Hickman et al., 2018). In this specimen, the microglia showed no activated forms and were evenly distributed, indicating a normal state (Figure 1A). CP13, a phospho-tau antibody that stains against the Serine 202 epitope, was applied to this specimen as well. Tau accumulates in dying neurons and can be found in pathological structures such as neurofibrillary tangles and neuritic clusters in cases of neurodegeneration, including AD (Spillantini and Goedert, 2013). This specimen did not show any neurofibrillary tangles or neuritic clusters, however, some neurons located at the lower margin of the cortical layer III stained positively for tau, as did some pretangles in layer II (Figure 1B). This distribution is similar to what would be expected in a middle-aged human, as age-related tau accumulation begins in layers II and III and progresses towards the deeper layers as neurodegeneration progresses (Moloney et al., 2021). The tissue was also stained with a combination of CP13 and glial fibrillary acidic protein (GFAP) using immunofluorescence. GFAP is expressed by astrocytes and the combination of activated astrocytes and tau-stained neurons is an indicator of neurodegeneration. In this specimen there were no activated astrocyte forms and no clumping around the tau-positive neurons. The tau “speckling” throughout the cortex (Figures 1B–F) may reflect axonal damage, or it may be a species-specific artifact. It could also be representative of early aging, and some similar (albeit less widespread) patterns appear in early-aging humans (Giannakopoulos et al., 2007; Tsartsalis et al., 2018). Investigations regarding signs of neurodegeneration are ongoing in other elephant specimens and species for a better understanding of how the elephant brain changes with age.

**FIGURE 2:**
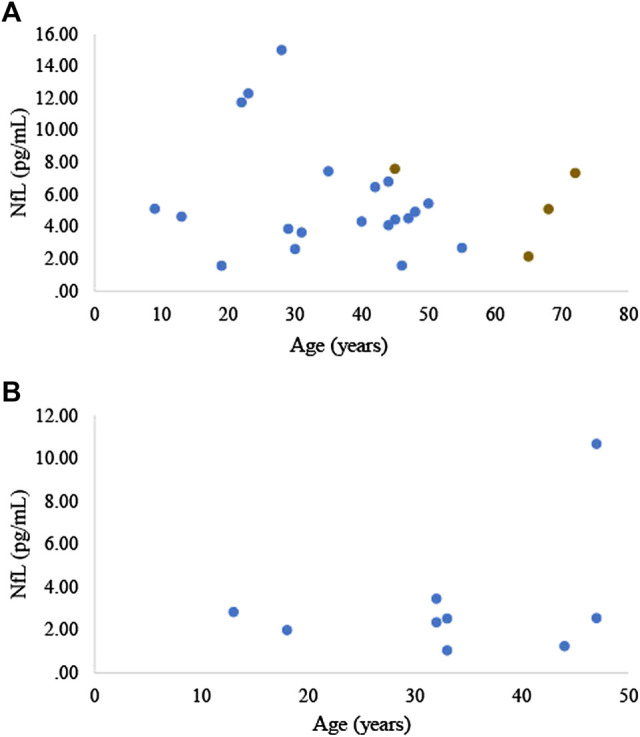
**(A)** Serum and **(B)** plasma NfL concentrations in zoo Asian elephants (*Elephas maximus*, n = 21; n = 9, respectively). The four brown data points represent samples collected from the same elephant.

Neurofilament light (NfL) is a highly phosphorylated neuronal structural protein that upon neuro-axonal damage is released into the extracellular space, and subsequently into the cerebrospinal fluid and blood (Holt-Lunstad et al., 2010). Numerous reports have been made of the association between serum and plasma NfL and the severity of acute central nervous system injury, as well as the presence and state of neurodegenerative disease, including AD in humans (Preische et al., 2019; Ashton et al., 2019), AD rodent models (Bacioglu et al., 2016; Andersson et al., 2020), and cognitive dysfunction in dogs (Panek et al., 2020). To our knowledge we are the first to measure NfL, or any neurodegenerative biomarker, in elephants. We measured serum NfL in 21 zoo Asian elephants (20 females, 1 male; 39.6 ± 16.1 years of age, range 9–72 years of age) and plasma NfL in 9 zoo Asian elephants (7 females, 2 males; 33.2 ± 11.3 years of age, range 13–47 years of age). NfL was analyzed using the Simoa NF-light digital immunoassay (103,186, Quanterix, Billerica, MA). Serum and plasma NfL concentrations averaged 5.6 ± 3.3 and 3.2 ± 2.7 pg/ml, respectively (Figures 2A,B). In one elephant, for which we had repeated serum samples collected at ages 45, 65, 68, and 72 years (Figure 2A), we did not observe a general increase in NfL concentrations over time. Using R statistical software (R version, 3.5.2), based on a linear mixed model for the serum samples and a linear regression model for the plasma samples, we did not find a significant relationship between NfL concentrations and age (*p* = 0.275; 0.341, respectively; significance level was determined at *p* < 0.05, 2-tailed). It is difficult to directly compare blood NfL concentrations across species as peripheral factors, such as body mass index, which can affect blood volume and reduce NfL levels, and kidney function, which affects protein clearance, can differ across species. Regardless, NfL concentrations obtained from zoo Asian elephants in either plasma or serum are lower than humans [e.g., see (Khalil et al., 2020)] and dogs (Panek et al., 2020). Further data are needed to determine whether elephants are protected to a certain degree against neurodegeneration.

## Conclusion

Despite the phylogenetic distance between elephants and humans, convergence in the evolution of longevity, sociality, cognition, and memory makes elephants an intriguing species for comparative investigation. Yet elephants have largely been overlooked as an animal model that could shed light on the diseases of aging, including cancer, AD, and comorbidities associated with adverse early life experiences. Developing these resources offers great potential for future research. Comprehensive life history and medical records exist for elephants living under human care, either in zoological institutions or in semi-captive conditions in range countries [e.g., records for ∼9,600 Myanmar timber elephants captured or born after 1875 (Mar et al., 2001)]. This allows for retrospective analyses and adjustment for differences in life experiences and health status. An intriguing opportunity also exists to compare species living in different environments, e.g., captive versus semi-captive versus wild populations. Zoological institutions are not as controlled like a traditional laboratory setting, yet they are more artificial than in the wild. Semi-captive populations fall in the middle, such that semi-captive elephants have access to veterinary care and diet supplementation but can also roam and interact with wild elephant herds. These differences in environment and social access allow for a range of comparative studies within species to examine the possible effects of external factors on the biology of aging. Study of elephants offers a novel and valuable perspective to aging research.
